# 1,3-Dihydr­oxy-2-(hydroxy­meth­yl)propan-2-aminium 2,2-dichloro­acetate

**DOI:** 10.1107/S1600536809016626

**Published:** 2009-05-14

**Authors:** Yan-Hong Yu, Kun Qian

**Affiliations:** a Jiangxi Key Laboratory of Organic Chemistry, Jiangxi Science and Technology Normal University, Nanchang 330013, People’s Republic of China; b Academic Administration of JiangXi University of Traditional Chinese Medicine, Nanchang 330047, People’s Republic of China

## Abstract

The title compound, C_4_H_12_NO_3_^+^·C_2_HCl_2_O_2_^−^, was obtained from dichloro­acetic acid and 2-amino-2-(hydroxy­meth­yl)propane-1,3-diol. In the crystal structure, the cations and anions are connected by inter­molecular N—H⋯O and O—H⋯O hydrogen bonding, forming a two-dimensional array parallel to (001). The crystal used for analysis was a merohedral twin, as indicated by the Flack parameter of 0.67 (6).

## Related literature

For the engineering of organic crystals for quadratic non-linear optics, see: Etter & Frankenbach (1989 ▶); Yaghi *et al.* (1997 ▶). For hydrogen-bond networks, see: Etter (1990 ▶).
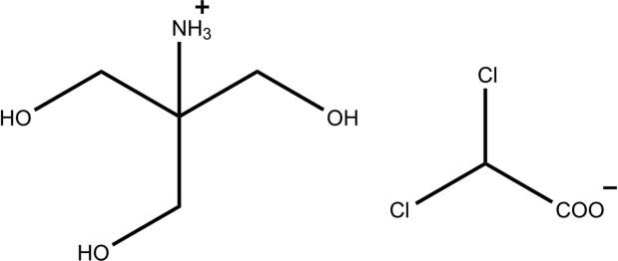


## Experimental

### 

#### Crystal data

C_4_H_12_NO_3_^+^·C_2_HCl_2_O_2_^−^
*M* *_r_* = 250.07
Monoclinic, 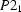

*a* = 8.6231 (17) Å
*b* = 6.1376 (12) Å
*c* = 9.898 (2) Å
β = 97.03 (3)°
*V* = 519.92 (18) Å^3^
*Z* = 2
Mo *K*α radiation
μ = 0.62 mm^−1^
*T* = 293 K
0.22 × 0.18 × 0.12 mm

#### Data collection

Rigaku SCXmini diffractometer
Absorption correction: multi-scan (*CrystalClear*; Rigaku, 2005 ▶) *T*_min_ = 0.875, *T*_max_ = 0.929
4914 measured reflections
2044 independent reflections
1951 reflections with *I* > 2σ(*I*)
*R*_int_ = 0.025

#### Refinement

*R*[*F* ^2^ > 2σ(*F* ^2^)] = 0.031
*wR*(*F* ^2^) = 0.069
*S* = 1.10
2044 reflections
130 parameters
3 restraints
H-atom parameters constrained
Δρ_max_ = 0.23 e Å^−3^
Δρ_min_ = −0.25 e Å^−3^
Absolute structure: Flack (1983 ▶), 920 Friedel pairs
Flack parameter: 0.67 (6)

### 

Data collection: *CrystalClear* (Rigaku, 2005 ▶); cell refinement: *CrystalClear*; data reduction: *CrystalClear*; program(s) used to solve structure: *SHELXS97* (Sheldrick, 2008 ▶); program(s) used to refine structure: *SHELXL97* (Sheldrick, 2008 ▶); molecular graphics: *ORTEPIII* (Burnett & Johnson, 1996 ▶), *ORTEP-3 for Windows* (Farrugia, 1997 ▶) and *PLATON* (Spek, 2009 ▶); software used to prepare material for publication: *SHELXL97*.

## Supplementary Material

Crystal structure: contains datablocks I, global. DOI: 10.1107/S1600536809016626/dn2442sup1.cif

Structure factors: contains datablocks I. DOI: 10.1107/S1600536809016626/dn2442Isup2.hkl

Additional supplementary materials:  crystallographic information; 3D view; checkCIF report

## Figures and Tables

**Table 1:** Hydrogen-bond geometry (Å, °)

*D*—H⋯*A*	*D*—H	H⋯*A*	*D*⋯*A*	*D*—H⋯*A*
N1—H1*A*⋯O3^i^	0.89	2.00	2.881 (2)	169
N1—H1*B*⋯O2^ii^	0.89	1.97	2.858 (2)	172
N1—H1*C*⋯O5^iii^	0.89	2.03	2.909 (2)	169
O3—H3⋯O4^iv^	0.81	1.85	2.654 (2)	169
O4—H4⋯O1	0.82	1.84	2.655 (2)	173
O5—H5⋯O2^v^	0.82	1.88	2.691 (2)	168

Symmetry codes: (i) 
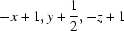
; (ii) 
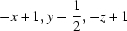
; (iii) 
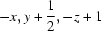
; (iv) 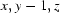; (v) 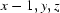.

**Fig. 1.**
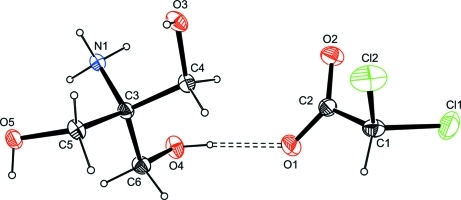
The molecular structure of the title compound with the atom labeling scheme. Displacement ellipsoids are drawn at the 30% probability level. H atoms are represented as small spheres of arbitrary radii. H bond is drawn as dashed line.

**Fig. 2.**
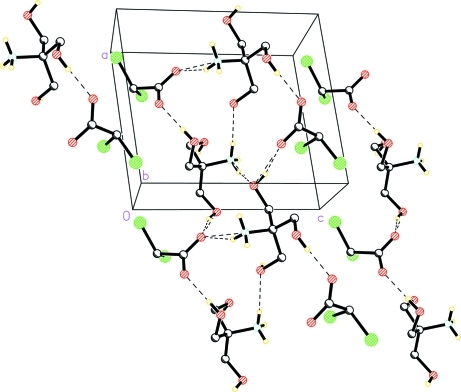
Partial packing view showing the intricated hydrogen bond framework. H atoms not involved in hydrogen bondings were omitted. [Symmetry code: (i) -x + 1, y + 1/2, -z + 1.]

## supplementary crystallographic information

### Comment

During the past 15 years, organic crystals for quadratic nonlinear optics have been intensely engineered (Etter & Frankenbach, 1989; Yaghi *et al.*, 1997). Arising from the complexation of organic and inorganic molecules based on acid–base interactions, highly polarisable cations, responsible for NLO properties, are linked to inorganic anions through hydrogen bond networks which generate a noncentrosymmetric structural organization (Etter, 1990). In this paper, a novel nonlinear hybrid molecular crystal, NH_2_C(CH_2_OH)_3_, has been prepared by complexation between dichloroacetic and tris(hydroxymethyl)amino methane.

The structure is built up from cations and anions (Fig. 1) connected through strong intermolecular hydrogen bonds (Table 1, Fig. 2) to form a two-dimensional layer developing parallel to the (001) plane. As suggested by the value of the Flack parameter (Flack, 1983), 0.67 (6), based on 920 Friedel's pairs, the particular crystal is twinned by inversion.

### Experimental

The crystals were grown by slow evaporation at ambient temperature of the solution prepared by adding dichloroacetic acid to the aqueous solution of tris(hydroxymethyl)aminomethane in a stoichiometric ratio. For the X-ray diffraction analysis, suitable single crystals of compound (I) were obtained after one night by slow evaporation from an filtration water solution.

### Refinement

All H atoms were found from a difference Fourier map but they were treated as riding on their parent atoms with C—H = 0.97 Å (methylene) or 0.98 Å (methine), N—H = 0.89 Å and O—H = 0.82 Å with *U*iso(H) = 1.2*U*eq(C) and *U*iso(H) = 1.5*U*eq(N,O).

### Crystal data

**Table:**

C_4_H_12_NO_3_^+^·C_2_HCl_2_O_2_^−^	*F*(000) = 260
*M**_r_* = 250.07	*D*_x_ = 1.597 Mg m^−^^3^
Monoclinic, *P*2_1_	Mo *K*α radiation, λ = 0.71073 Å
Hall symbol: P 2yb	Cell parameters from 735 reflections
*a* = 8.6231 (17) Å	θ = 2.8–27.5°
*b* = 6.1376 (12) Å	µ = 0.62 mm^−^^1^
*c* = 9.898 (2) Å	*T* = 293 K
β = 97.03 (3)°	Prism, colourless
*V* = 519.92 (18) Å^3^	0.22 × 0.18 × 0.12 mm
*Z* = 2	

### Data collection

**Table:**

Rigaku SCXmini diffractometer	2044 independent reflections
Radiation source: fine-focus sealed tube	1951 reflections with *I* > 2σ(*I*)
graphite	*R*_int_ = 0.025
Detector resolution: 13.6612 pixels mm^-1^	θ_max_ = 26.0°, θ_min_ = 3.3°
ω scans	*h* = −10→10
Absorption correction: multi-scan (*CrystalClear*; Rigaku, 2005)	*k* = −7→7
*T*_min_ = 0.875, *T*_max_ = 0.929	*l* = −12→12
4914 measured reflections	

### Refinement

**Table:**

Refinement on *F*^2^	Secondary atom site location: difference Fourier map
Least-squares matrix: full	Hydrogen site location: inferred from neighbouring sites
*R*[*F*^2^ > 2σ(*F*^2^)] = 0.031	H-atom parameters constrained
*wR*(*F*^2^) = 0.069	*w* = 1/[σ^2^(*F*_o_^2^) + (0.0218*P*)^2^ + 0.166*P*] where *P* = (*F*_o_^2^ + 2*F*_c_^2^)/3
*S* = 1.10	(Δ/σ)_max_ < 0.001
2044 reflections	Δρ_max_ = 0.23 e Å^−^^3^
130 parameters	Δρ_min_ = −0.25 e Å^−^^3^
3 restraints	Absolute structure: Flack (1983), 920 Friedel pairs
Primary atom site location: structure-invariant direct methods	Flack parameter: 0.67 (6)

### Special details

**Table:**

Geometry. All esds (except the esd in the dihedral angle between two l.s. planes) are estimated using the full covariance matrix. The cell esds are taken into account individually in the estimation of esds in distances, angles and torsion angles; correlations between esds in cell parameters are only used when they are defined by crystal symmetry. An approximate (isotropic) treatment of cell esds is used for estimating esds involving l.s. planes.
Refinement. Refinement of *F*^2^ against ALL reflections. The weighted *R*-factor *wR* and goodness of fit *S* are based on *F*^2^, conventional *R*-factors *R* are based on *F*, with *F* set to zero for negative *F*^2^. The threshold expression of *F*^2^ > σ(*F*^2^) is used only for calculating *R*-factors(gt) *etc*. and is not relevant to the choice of reflections for refinement. *R*-factors based on *F*^2^ are statistically about twice as large as those based on *F*, and *R*- factors based on ALL data will be even larger.

### Fractional atomic coordinates and isotropic or equivalent isotropic displacement parameters (Å^2^)

**Table:**

	*x*	*y*	*z*	*U*_iso_*/*U*_eq_	
Cl1	0.86491 (9)	0.39154 (15)	0.00483 (7)	0.0630 (2)	
Cl2	0.70850 (10)	0.03942 (11)	0.13016 (8)	0.0598 (2)	
C1	0.6938 (3)	0.3135 (4)	0.0737 (2)	0.0342 (5)	
H1	0.6046	0.3263	0.0026	0.041*	
C2	0.6675 (2)	0.4656 (4)	0.1919 (2)	0.0282 (5)	
C3	0.2265 (2)	0.2569 (3)	0.3756 (2)	0.0215 (4)	
C4	0.3748 (2)	0.1471 (3)	0.3394 (2)	0.0248 (4)	
H4A	0.3540	0.0827	0.2496	0.030*	
H4B	0.4558	0.2562	0.3365	0.030*	
C5	0.0883 (2)	0.0986 (3)	0.3577 (2)	0.0249 (4)	
H5A	0.0543	0.0801	0.2613	0.030*	
H5B	0.1226	−0.0423	0.3944	0.030*	
C6	0.1911 (2)	0.4607 (3)	0.2892 (2)	0.0270 (4)	
H6A	0.1841	0.4215	0.1937	0.032*	
H6B	0.0905	0.5188	0.3058	0.032*	
N1	0.25465 (19)	0.3243 (3)	0.52217 (16)	0.0221 (3)	
H1A	0.3470	0.3903	0.5382	0.033*	
H1B	0.2540	0.2070	0.5750	0.033*	
H1C	0.1797	0.4156	0.5403	0.033*	
O1	0.54454 (19)	0.5709 (3)	0.17435 (18)	0.0444 (4)	
O2	0.76863 (19)	0.4708 (3)	0.29336 (16)	0.0403 (4)	
O3	0.42905 (16)	−0.0160 (2)	0.43407 (15)	0.0306 (4)	
H3	0.3857	−0.1281	0.4071	0.046*	
O4	0.30642 (17)	0.6233 (2)	0.31776 (17)	0.0336 (4)	
H4	0.3805	0.5959	0.2759	0.050*	
O5	−0.04042 (15)	0.1704 (3)	0.42311 (15)	0.0274 (3)	
H5	−0.0931	0.2564	0.3735	0.041*	

### Atomic displacement parameters (Å^2^)

**Table:**

	*U*^11^	*U*^22^	*U*^33^	*U*^12^	*U*^13^	*U*^23^
Cl1	0.0535 (4)	0.0984 (7)	0.0405 (4)	−0.0075 (4)	0.0196 (3)	−0.0042 (4)
Cl2	0.0872 (5)	0.0321 (3)	0.0549 (4)	−0.0001 (4)	−0.0123 (4)	−0.0081 (3)
C1	0.0339 (12)	0.0377 (13)	0.0292 (11)	0.0008 (10)	−0.0040 (9)	−0.0007 (10)
C2	0.0274 (11)	0.0271 (10)	0.0304 (11)	−0.0002 (10)	0.0048 (9)	0.0064 (9)
C3	0.0185 (9)	0.0225 (10)	0.0233 (10)	−0.0014 (8)	0.0018 (8)	0.0011 (8)
C4	0.0213 (10)	0.0248 (11)	0.0288 (11)	0.0005 (9)	0.0052 (9)	0.0000 (9)
C5	0.0191 (9)	0.0233 (11)	0.0324 (11)	−0.0007 (8)	0.0029 (8)	−0.0030 (9)
C6	0.0247 (10)	0.0227 (10)	0.0332 (11)	0.0007 (9)	0.0017 (9)	0.0044 (9)
N1	0.0180 (7)	0.0224 (8)	0.0259 (9)	0.0000 (7)	0.0031 (7)	−0.0006 (7)
O1	0.0347 (9)	0.0503 (11)	0.0494 (10)	0.0142 (8)	0.0097 (8)	0.0129 (9)
O2	0.0424 (9)	0.0425 (10)	0.0337 (9)	0.0120 (8)	−0.0048 (7)	−0.0105 (7)
O3	0.0238 (7)	0.0236 (8)	0.0433 (9)	0.0036 (6)	−0.0002 (7)	0.0001 (7)
O4	0.0293 (8)	0.0219 (7)	0.0510 (10)	−0.0038 (6)	0.0105 (7)	0.0024 (7)
O5	0.0179 (7)	0.0308 (8)	0.0337 (8)	−0.0002 (6)	0.0043 (6)	0.0027 (6)

### Geometric parameters (Å, °)

**Table:**

Cl1—C1	1.765 (2)	C5—O5	1.421 (2)
Cl2—C1	1.773 (3)	C5—H5A	0.9700
C1—C2	1.536 (3)	C5—H5B	0.9700
C1—H1	0.9800	C6—O4	1.413 (3)
C2—O1	1.236 (3)	C6—H6A	0.9700
C2—O2	1.247 (3)	C6—H6B	0.9700
C3—N1	1.499 (3)	N1—H1A	0.8900
C3—C6	1.525 (3)	N1—H1B	0.8900
C3—C4	1.527 (3)	N1—H1C	0.8900
C3—C5	1.531 (3)	O3—H3	0.8119
C4—O3	1.411 (2)	O4—H4	0.8205
C4—H4A	0.9700	O5—H5	0.8200
C4—H4B	0.9700		
			
C2—C1—Cl1	109.75 (16)	O5—C5—C3	112.99 (16)
C2—C1—Cl2	110.36 (16)	O5—C5—H5A	109.0
Cl1—C1—Cl2	110.39 (14)	C3—C5—H5A	109.0
C2—C1—H1	108.8	O5—C5—H5B	109.0
Cl1—C1—H1	108.8	C3—C5—H5B	109.0
Cl2—C1—H1	108.8	H5A—C5—H5B	107.8
O1—C2—O2	127.1 (2)	O4—C6—C3	112.27 (17)
O1—C2—C1	114.5 (2)	O4—C6—H6A	109.2
O2—C2—C1	118.38 (19)	C3—C6—H6A	109.2
N1—C3—C6	108.33 (17)	O4—C6—H6B	109.2
N1—C3—C4	107.93 (16)	C3—C6—H6B	109.2
C6—C3—C4	110.28 (16)	H6A—C6—H6B	107.9
N1—C3—C5	108.57 (16)	C3—N1—H1A	109.5
C6—C3—C5	110.83 (16)	C3—N1—H1B	109.5
C4—C3—C5	110.81 (17)	H1A—N1—H1B	109.5
O3—C4—C3	112.12 (16)	C3—N1—H1C	109.5
O3—C4—H4A	109.2	H1A—N1—H1C	109.5
C3—C4—H4A	109.2	H1B—N1—H1C	109.5
O3—C4—H4B	109.2	C4—O3—H3	106.3
C3—C4—H4B	109.2	C6—O4—H4	109.1
H4A—C4—H4B	107.9	C5—O5—H5	109.5

### Hydrogen-bond geometry (Å, °)

**Table:**

*D*—H···*A*	*D*—H	H···*A*	*D*···*A*	*D*—H···*A*
N1—H1A···O3^i^	0.89	2.00	2.881 (2)	169
N1—H1B···O2^ii^	0.89	1.97	2.858 (2)	172
N1—H1C···O5^iii^	0.89	2.03	2.909 (2)	169
O3—H3···O4^iv^	0.81	1.85	2.654 (2)	169
O4—H4···O1	0.82	1.84	2.655 (2)	173
O5—H5···O2^v^	0.82	1.88	2.691 (2)	168

Symmetry codes: (i) −*x*+1, *y*+1/2, −*z*+1; (ii) −*x*+1, *y*−1/2, −*z*+1; (iii) −*x*, *y*+1/2, −*z*+1; (iv) *x*, *y*−1, *z*; (v) *x*−1, *y*, *z*.
